# Gleanings

**Published:** 1896-11

**Authors:** 


### GLEANINGS.

THE use of Fowler's or Donovan's solution of arsenic for warts is highly commended in human practice, and might well be tried in equine practice, where these blemishes of the cuticle are very much of an eyesore.

The recent adoption of asbestos as a surgical dressing has one very excellent point to commend it, in that it can be exposed to a degree of heat sufficient to destroy all germ-life.

A band of wild horses, the only ones thought to be in the territories, were recently sighted on the borderlands between the United States and Mexico. They are estimated at about eighty in number.

The purifying of whiskey and elimination of all deleterious matter by electrical process, at a nominal cost, is one of the latest inventions.

A Spanish commission for the purchase of 2000 broncho ponies was recently issued, the ponies to be used in the Cuban rebellion by the Spanish soldiers. We have no doubt of their proving more than satisfactory for such purposes, as they are well adapted for hardship and privation.

The *Poultry-Keeper* recommends the following mixture for lice on fowls and other animals: One and a half gallons of kerosene are soaked through two and a half pounds of pyrethrum (Persian powder), forming a yellow oily extract. Dissolve one pound of soap in one gallon of the extract and churn until thoroughly emulsified. When using, mix one pint of the emulsion with four pints of water.

A silo capable of feeding 350 cows, fifty pounds each a day for three hundred and sixty-five days, has been constructed at Aztalan, Wisconsin.

An ex-army veterinarian failed to transmit strangles, or rhino-adenitis, to goats and dogs, with introduction of lancet on which fresh pus was secured. He reports that Indians eating the flesh of animals dying from distemper appeared to suffer no inconvenience therefrom.

